# Sputum Gram stain for diagnosing causative bacterial pathogens and guiding antimicrobial therapies in community-acquired pneumonia: a systematic review and meta-analysis protocol

**DOI:** 10.20407/fmj.2018-019

**Published:** 2019-04-17

**Authors:** Hiroaki Ogawa, Georgios D. Kitsios, Mitsunaga Iwata, Teruhiko Terasawa

**Affiliations:** 1 Department of Emergency and General Internal Medicine, Fujita Health University, School of Medicine, Toyoake, Aichi, Japan; 2 Division of Pulmonary, Allergy and Critical Care Medicine, University of Pittsburgh, PA, USA

**Keywords:** Community-acquired pneumonia, Gram stain, Meta-analysis

## Abstract

**Objectives:**

The clinical role of sputum Gram stain for rapid etiologic pathogen diagnosis in patients with community-acquired pneumonia (CAP) remains an unresolved controversy. Variability in protocols and reporting of diagnostic performance in different studies has hampered assessments of clinical utility and interpretation. Since the last meta-analysis published in 1996, several reports and resources to accurately evaluate the diagnostic accuracy of sputum Gram stain have become available. Therefore, we will conduct a systematic review and meta-analysis of the clinical validity and utility of sputum Gram stain.

**Methods:**

We will search PubMed, Ovid MEDLINE, Embase, and The Cochrane Controlled Register of Trials (CENTRAL) databases from inception through July 30, 2018, with no language restriction and perform a full-text evaluation of potentially relevant articles. We will include prospective and retrospective studies that assess sputum Gram stain in adults (aged ≥18 years) with CAP. Two reviewers will independently extract data and rate each study’s validity with standard quality assessment tools. We will subsequently perform standard and latent-class random-effects model meta-analyses to quantitatively synthesize the diagnostic accuracy and yield. Finally, we will assess the totality of evidence by the Grading of Recommendations Assessment, Development, and Evaluation (GRADE) approach for diagnostic tests and strategies.

**Results:**

Results of the analysis will be submitted for publication in a peer-reviewed journal.

**Conclusions:**

This systematic review and meta-analysis will provide a 30-year synopsis of clinical evidence on sputum Gram stain in patients with CAP.

## Introduction

Community-acquired pneumonia (CAP) is an acute infection of the lung that develops in community-dwelling persons who have not been hospitalized in recent months or have not received regular medical or nonmedical healthcare.^1^ Despite advances in effective antimicrobial therapies, lower respiratory tract infections were the fourth most common cause of death and the leading infectious cause of death worldwide in 2016.^2^ Although mortality from pneumonia has been decreasing in the United States, with 50,000 annual deaths in 2015, approximately 1 million adults were hospitalized with pneumonia in the same year, making it the second most common cause of admissions in the United States.^3^ In Japan, pneumonia remains the third most common cause of death, and approximately 120,000 people died of pneumonia in 2015.^4^

Gram staining of expectorated sputum is a simple, easily performed, widely available, and inexpensive test for patients with pneumonia. Multiple pathogens can be assessed simultaneously with sputum Gram stain, and the test has a short turnaround time.^5^ When performed by experienced observers with specimens of acceptable quality, the sputum Gram stain can assist in establishing the correct pathogen diagnosis in CAP and in directing appropriate antibiotic therapies. However, the wide variability in reported sensitivity and specificity of heterogeneously conducted studies has led to inconsistent adoption of the test in clinical practice.^6^ In addition, inadequate sputum samples are not uncommon, and processing of the specimens and microscopic diagnosis of causative bacteria by visual assessment are highly operator-dependent.^7^ Other factors, such as collection and transport of the specimens, can affect timely initiation of antimicrobial therapies, which is an important measure related to pneumonia-associated mortality.^8^ Furthermore, to our knowledge, no robust evidence exists to support a pathogen-directed treatment strategy over the guideline-recommended empirical broad-spectrum antibiotic treatment.^9^ Therefore, current clinical guidelines for patients with CAP inconsistently recommend sputum Gram stain only in selected indications.^10–12^ Nevertheless, a theoretical rationale for pathogen-directed therapies was that rapid detection of pneumonia etiologies could spare the use (and misuse) of broad-spectrum antibiotics to control the emergence of antibiotic resistance.

In the past two decades since the publication of the meta-analysis of sputum Gram stain in patients with CAP in 1996,^6^several new primary studies have assessed this topic. Before introduction of contemporary microbiological tests, such as antigen testing for *Streptococcus pneumoniae*, *Mycoplasma pneumoniae*, *Legionella pneumophila*, and influenza virus, and nuclear acid amplification tests for bacteria and other pathogens, including viruses and *M. pneumoniae*, *Chlamydophila pneumoniae*, and *L. pneumophila*, primary studies had to rely on less sensitive culture-based reference standards. Diagnostic accuracy of an index test is, in theory, biased when the reference standard is imperfect, and the direction of the bias depends on whether the index and reference tests are dependent (or independent).^13^ For this reason, several approaches to account for the imperfectness of reference standards have been implemented to calculate corrected accuracy in primary studies.^14^ Thus, the naïvely synthesized uncorrected accuracy estimates in the 1996 meta-analysis could be inaccurate. Furthermore, the 1996 meta-analysis focused on diagnostic accuracy for detecting *Strep. pneumoniae* only and failed to include diagnostic accuracy for detecting other potentially important pathogens or the overall diagnostic yield to assess the full range of performance in this modality to simultaneously assess multiple pathogens.

Given the emergence of the aforementioned contemporary reference standard and alternative or add-on tests and the approaches available that adjust for theoretically biased results, we plan a comprehensive overview and quantitative synthesis of the clinical data on sputum Gram stain for identifying causative pathogens of CAP.

## Methods

This systematic review and meta-analysis protocol follows the preferred reporting items for systematic review and meta-analysis protocols 2015 statement (PRISMA-P).^15^

We have followed the framework for assessing levels of clinical effectiveness of diagnostic tests proposed by Fryback and Thornbury^16^ and formulated the following five research questions:

Research Question 1 (diagnostic accuracy; Fryback Level 2):

What is the diagnostic accuracy of sputum Gram stain (alone or in combination with other tests, such as *Strep. pneumoniae* antigen testing of the urine; separately analyzed) to diagnose the following specific pathogens for patients with CAP?

1.  *Strep. pneumoniae*
2.  *Haemophilus influenzae*
3.  *Klebsiella pneumoniae*
4.  *Moraxella catarrhalis*
5.  *Pseudomonas aeruginosa*
6.  *Staphylococcus aureus*

We chose, *a priori*, these six common, clinically significant, and morphologically discernible bacterial pathogens of CAP as the target pathogens;^5^ previous studies^9,17^ and guidelines^18^ have proposed specific antibiotics for these six bacteria. We will also assess a combined pathogen category, named “mixed (aerobic and anaerobic) oral flora,” as a target pathogen of interest.

Research Question 2 (diagnostic impact; Fryback Level 3):

What is the proportion of patients for whom sputum Gram stain (alone or in combination; separately analyzed) is useful in diagnosing specific pathogens for patients with CAP?

Research Question 3 (management decision impact; Fryback Level 4):

How often does sputum Gram stain (alone or in combination; separately analyzed) change diagnostic or therapeutic strategies planned before testing for patients with CAP?

Research Question 4 (patient-relevant outcomes; Fryback Level 5):

What is the comparative effectiveness between diagnostic and management strategies guided by sputum Gram stain and those not guided by sputum Gram stain for patients with CAP? In this question, we will, assess as specific patient-relevant outcomes, (1) failure rates of primary therapies, (2) overall failure rates of primary and subsequent-line therapies, (3) length of hospital stay, (4) in-hospital mortality, and (5) all harms observed. Here, we will define failure as a patient whose signs and symptoms of pneumonia do not improve within a study-specified time-frame after initiation of the initial antibiotic treatment and is therefore judged a “treatment failure” by the study.

Research Question 5 (effect modifiers):

What factors modify the aforementioned effectiveness measures?

### Information sources and search strategies

We will search PubMed and MEDLINE, Embase, and the Cochrane Register of Controlled Trials (CENTRAL) via Ovid databases from inception through July 30, 2018, using the free-text terms “sputum,” “Gram stain,” and “pneumonia,” and their synonyms. As additional searches, we will peruse the reference lists of previously reported systematic reviews and meta-analyses. A pulmonologist investigator who specializes in pneumonia microbiome (GDK) will check whether there are any missing publications that are relevant. No language restrictions will be set.

### Eligibility criteria

We will include any prospective or retrospective cohort or cross-sectional studies that included at least ten patients with CAP and that assessed the outcomes of interest listed under the PICO framework (Table 1). We will also include randomized controlled trials and non-randomized studies of intervention of any size that assessed the effectiveness of sputum Gram stain in patients with CAP (e.g., test-directed versus no test strategies).

We will exclude conference abstracts, primary studies with the outcome data unextractable from the publication, and studies based on modeling without using primary data.

Two reviewers will independently screen abstracts, and all potentially eligible articles considered by at least one reviewer will be retrieved. Then, the two reviewers will independently peruse the retrieved full-text articles and determine the final inclusion. Any discrepant results will be resolved by consensus. Adjudication by a third reviewer will be made in case of unresolved discrepancies.

### Data extraction

Data will be extracted by two reviewers. One primary reviewer will extract the following descriptive data, and (at least) one reviewer will verify all extracted data. Two independent reviewers will extract any numerical data on the outcomes of interest. Disagreements will be resolved by consensus including a third reviewer.

In the case of missing or unresolved numerical data, we will contact the study authors for clarification by email. We will send two additional email correspondences if no response is received within 2 weeks of a previous correspondence attempt.

We will extract study, patient, and test characteristics as descriptive data. Study characteristics will include study identification, study location (country, city), study period (enrollment year), study design, enrollment methods (consecutive or not), number of centers, clinical setting, definition of CAP, exclusion criteria, comparator tests if any, and types of reference standard adopted. Patient characteristics will include number of patients, average age (range), male sex (%), presence of chronic obstructive pulmonary disease (%), immunocompromised host (%), suspected aspiration pneumonia (%), prognostic index (e.g., pneumonia severity index), prior antibiotics use, identified pathogens [*Strep. pneumoniae* (%), *H. influenzae* (%), *M. catarrhalis* (%), *K. pneumoniae* (%), *P. aeruginosa* (%), *Staph. aureus* (%), mixed oral flora (%)], non-pneumonia causes (%), and unidentified pathogens or causes (%). Test characteristics will include timing of sampling, sampling methods, time between sampling and Gram stain, staining methods, validity criteria of adequate samples, adequate samples [*n*/*N* (%)], performers of Gram stain and experience, and interpreter of test results and experience.

### Primary and secondary outcomes and definitions of the outcome measures

We will assess sensitivity and specificity as the outcome measure of diagnostic accuracy (the primary outcome of interest). We will define sensitivity as TP/(TP+FN) and specificity as TN/(FP+TN), where TP indicates true-positive (positive index and reference standard tests), FP indicates false-positive (index test positive and reference standard test negative), FN indicates false-negative (index test negative and reference standard test positive), and TN indicates true-negative (index and reference standard tests negative) results from the 2×2 contingency table including cross-classified count data according to whether the index and reference standard tests are positive or negative. Here, we will consider the morphological visual assessment of each specific bacterium observed after Gram staining as the index test.

We will assess diagnostic yield as the measure of diagnostic impact (as the secondary outcome). We will define diagnostic yield as the number of cases with a correct diagnosis by testing (any correctly diagnosed bacteria by sputum Gram stain; this number should correspond to the total number of TP cases for all Gram stain–assessable bacteria) divided by the number of all tested cases. We will perform a subgroup analysis of diagnostic yield for patients with sputum samples of adequate quality.

As the measure of management decision impact, we will calculate the post-test percentage change in the diagnostic or therapeutic interventions planned before performing sputum Gram stain in a study cohort. The respective percentage changes will be defined as the number of patients for whom the diagnostic or therapeutic interventions planned before testing are altered based on the test results (regardless of whether the interventions are either increased or decreased) divided by the total number of patients who undergo sputum Gram stain.

Regarding the patient-relevant outcomes listed in Research Question 4, we will assess the association of use versus non-use of sputum Gram stain with the numbers of failure of antibiotic therapies, in-hospital deaths from any cause, and all harms observed as the binary outcomes; or length of hospital stay as the continuous outcome. For each study, we will calculate the risk ratio for each of the binary outcomes and the difference in length of hospital stay as the respective outcome measure.

### Reference standards

We will accept any reference standard test adopted in eligible studies. However, before analysis, we will specify commonly available clinical tests for each target pathogen as the reference standards and will define their results to uniformly construct the 2×2 table. Table 2 describes the operational definitions of the final diagnosis for the target pathogens by the reference standard tests.

### Assessment of risk of bias

To assess the risk of bias and concerns regarding the applicability of studies of diagnostic accuracy and yield, two reviewers will independently assess patient selection, index test, reference standard, and their flow and timing based on the revised Quality Assessment of Diagnostic Accuracy Studies instrument tool (QUADAS-2).^21^ Discrepant ratings will be resolved by consensus.

For non-randomized studies of intervention, we will use the ROBINS-I tool [Risk Of Bias In Non-randomized Studies - of Interventions, formerly known as A Cochrane Risk Of Bias Assessment Tool for Non-Randomized Studies of Interventions (ACROBAT-NRSI)], a recently proposed risk of bias assessment tool by the Cochrane Risk of Bias Group.^22^ For randomized controlled trials, we will use the revised tool to assess risk of bias in randomized trials (RoB 2 tool).^23^

We will rate each methodological quality item as “yes,” “no,” or “unclear” (due to no or less clear reporting) for each eligible study. Then, we will rate the overall validity for each study as being of low, intermediate, or high risk of bias.

### Data synthesis

For each specific pathogen, we will calculate sensitivity and specificity for each study with their corresponding 95% confidence intervals (CI) and then obtain summary estimates of sensitivity and specificity with their corresponding 95% CI by using bivariate random-effects meta-analysis with the exact binomial likelihood when ≥4 studies are available.^24,25^ We will assess between-study heterogeneity visually by plotting sensitivity and specificity separately in forest plots and in the receiver operating characteristic (ROC) space. We will construct hierarchical summary ROC curves (HSROC)^26^ and confidence regions for summary sensitivity and specificity when appropriate.^24,25^

We will calculate “adjusted” summary estimates of sensitivity and specificity, and summary ROC curves by a Bayesian latent-class model (LCM) meta-analysis to adjust for imperfect reference standard(s), as proposed by Dendukuri.^27^ In the main analysis, we will use a vague prior distribution (0%–100%) for the sensitivity and specificity of all adopted imperfect reference standard(s). In sensitivity analysis, we will use informative prior distributions for specific reference standard(s) adopted. For example, we will use a sensitivity of 74.0% (range, 66.6%–82.3%) and specificity of 97.2% (range, 92.7%–99.8%) for urine-based pneumococcal antigen tests based on the ranges reported in a meta-analysis accounting for the imperfect reference standard.^28^ However, a pulmonologist investigator who specializes in pneumonia microbiome (GDK) will propose clinically relevant ranges of accuracy estimates for any imperfect reference standard tests adopted in the primary studies.

Regarding the change in diagnosis, diagnostic or therapeutic managements, and patient-relevant outcomes, we will first perform qualitative syntheses through graphs and tables. If feasible, we will then calculate summary estimates of diagnostic yield and percentage change in diagnostic and therapeutic management by the random-effects meta-analysis of proportions, and summary risk ratios and differences by the standard Bayesian hierarchical random-effects meta-analysis.^29,30^

### Additional analyses

We will perform subgroup or univariable meta-regression analysis on study year (before versus after 2000), study location (United States and Europe versus other regions), use of a urine-based *Pneumococcus* test as reference standard (yes versus no), and performers/interpreter of test (physicians versus lab technicians; experienced personnel versus less experienced personnel). We will also assess the relationship between diagnostic yield and the prevalence of *Strep. pneumoniae* and *H. influenzae*, two of the most frequently identified pathogens for which sputum Gram stain is expected to be particularly useful. We will assess the totality of evidence by the Grading of Recommendations Assessment, Development, and Evaluation (GRADE) approach and strength of recommendations for diagnostic tests and strategies.^31^

We will not perform statistical tests for funnel plot asymmetry because the required tests do not allow for valid assessment of the extent and impact of missing data in studies of diagnostic accuracy.^32^ All aforementioned statistical analyses will be performed using Stata SE, version 13.1 (College Station, TX, USA) and WinBUGS 1.4.3 (MRC Biostatistics, Cambridge, UK) or OpenBUGS 3.2.3 (OpenBUGS Project Management Group; www.openbugs.net) from within Stata. All *P*-values will be two-sided, and statistical significance will be defined as *P*<0.05.

## Discussion

Sputum Gram stain is an inexpensive, readily available, and rapid test; together with other available rapid antigen detection tests, it is a pragmatic tool for rapid pathogen-directed antimicrobial therapy across different care settings. Although advanced sequencing-based molecular diagnostics are currently under development, such techniques remain investigational and have not been clinically validated.^33^ Thus, the diagnostic performance of sputum Gram staining and its impact on patient outcomes represent important and clinically relevant questions.

Use of the LCM meta-analysis has the potential to perform statistical corrections for the biased accuracy estimates reported in culture-based primary studies of sputum Gram stain in the absence of perfect reference standards, which is a strength of our analysis. Our comprehensive assessment of diagnostic accuracy and yield for all relevant bacterial pathogens also elucidates additional roles of sputum Gram stain, not limited to its role in diagnosing *Strep. pneumoniae*, in the management of CAP.

In conclusion, by conducting a 30-year field synopsis of this topic, including standard and LCM meta-analysis of diagnostic accuracy and yield, we hope to clarify the true diagnostic accuracy of sputum Gram stain for various bacterial pathogens on which further studies can be performed to address clinical impacts of pathogen-directed treatment strategies for patients with CAP.

## Figures and Tables

**Table1:** Inclusion criteria and clinical outcomes of interest based on the PICO framework

PICO	Specific details	Others
Population	Adult patients (aged ≥18 years) with community-acquired pneumonia	Per-study defined diagnostic criteria are allowed
Intervention test	Sputum Gram stain	Both self-expectorated and suctioned samples are allowed
Comparator/reference standard tests	• Sputum culture	Composite reference standards based on combinations of any adopted tests are allowed
	• Blood culture	
	•Antibodies for “atypical” pathogens, including non-viral causes such as *Mycoplasma pneumoniae*, *Chlamydophila pneumoniae*, and *Legionella pneumophila*, and viral causes such as respiratory syncytial virus, influenza virus, parainfluenza virus, adenovirus, and severe acute respiratory syndrome virus.	
	•Antigen tests (including point-of-care tests) for selected pathogens such as *Streptococcus pneumoniae*, *M. pneumoniae*, *L. pneumophila* (serotype 1 only), and influenza virus.	
	•Nuclear acid amplification tests for selected pathogens such as *M. pneumoniae*, *C. pneumoniae*, and *L. pneumophila*.	
	•Clinical follow-up including response to a specific treatment	
Outcomes	Fryback Level 2	
	•Test performance (e.g., sensitivity, specificity) using one or more above-listed tests and/or clinical follow-up as the reference standard	
	Fryback Level 3	
	•Change in diagnosis (diagnostic yield) and planned diagnostic approaches after obtaining test results	
	•Change in treatment plan after obtaining test results	
	Fryback Level 4	
	•Failure rates of primary therapy	
	•Overall failure rates of primary and subsequent-line therapies	
	•Delay in appropriate antibiotic use (e.g., time from presentation to definitive diagnosis; time from presentation to initiation of appropriate treatment)	
	•Length of hospital stay	
	•Intubation/mechanical ventilation	
	•Mortality (e.g., in-hospital or 30-day)	
	•Adverse effects of intervention(s) including direct harms of testing (e.g., airway bleeding or trauma due to suctioning); indirect harms of test-directed treatment (e.g., *Clostridium difficile* infection); and	
	•Direct cost (e.g., proportion of inadequate samples)	

**Table2:** Operational definitions of target pathogen positive and negative by clinical reference standards

Target pathogen	Pathogen positive	Pathogen negative
*Streptococcus pneumoniae*	*Strep. pneumoniae* detected by sputum culture, blood culture, or urine antigen test	Other pathogen(s) only detected by any microbiological tests* (possibility of co-infection still cannot be eliminated)
*Haemophilus influenzae*	*H. influenzae* detected by sputum culture or blood culture	
*Moraxella catarrhalis*	*M. catarrhalis* detected by sputum culture or blood culture	
*Klebsiella pneumoniae*	*K. pneumoniae* detected by sputum culture or blood culture	
*Pseudomonas aeruginosa*	*P. aeruginosa* detected by sputum culture or blood culture	
*Staphylococcus aureus*	*Staph. aureus* detected by sputum culture or blood culture	
Mixed (aerobic and anaerobic) oral flora	Mixed oral flora detected by sputum culture or blood culture	

*Sputum culture; blood culture; antibodies for “atypical” pathogens, including non-viral causes such as *Mycoplasma pneumoniae*, *Chlamydophila pneumoniae*, and *Legionella pneumophila*, and viral causes such as respiratory syncytial virus, influenza virus, parainfluenza virus, adenovirus, and severe acute respiratory syndrome virus; antigen tests (typically, point-of-care tests) for selected pathogens such as *Strep. pneumoniae*, *M. pneumoniae*, *L. pneumophila* (serotype 1 only), and influenza virus; nuclear acid amplification tests for selected pathogens such as *M. pneumoniae*, *C. pneumoniae*, and *L. pneumophila*.
